# Comparison of Simulated Outcomes Between Stool- and Blood-Based Colorectal Cancer Screening Tests

**DOI:** 10.1089/pop.2023.0037

**Published:** 2023-08-14

**Authors:** A. Mark Fendrick, Vahab Vahdat, Jing Voon Chen, David Lieberman, Paul J. Limburg, A. Burak Ozbay, John B. Kisiel

**Affiliations:** ^1^ Division of General Medicine, Departments of Internal Medicine and Health Management and Policy, University of Michigan, Ann Arbor, Michigan, USA.; ^2^ Exact Sciences Corporation, Madison, Wisconsin, USA.; ^3^ Division of Gastroenterology and Hepatology, School of Medicine, Oregon Health and Science University, Portland, Oregon, USA.; ^4^ Division of Gastroenterology and Hepatology, Mayo Clinic, Rochester, Minnesota, USA.

**Keywords:** colorectal neoplasms/prevention and control, liquid biopsy, biomarker, adenoma, Medicare

## Abstract

The Centers for Medicare & Medicaid Services (CMS) recommend covering blood-based tests meeting proposed minimum performance thresholds for colorectal cancer (CRC) screening. Outcomes were compared between currently available stool-based screening tests and a hypothetical blood-based test meeting CMS minimum thresholds. Using the Colorectal Cancer and Adenoma Incidence and Mortality Microsimulation Model (CRC-AIM), outcomes were simulated for average-risk individuals screened between ages 45 and 75 years with triennial multitarget stool DNA (mt-sDNA), annual fecal immunochemical test (FIT), and annual fecal occult blood test (FOBT). Per CMS guidance, blood-based CRC screening was modeled triennially, with 74% CRC sensitivity and 90% specificity. Although not specified by CMS, adenoma sensitivity was set between 10% and 20%. Published adenoma and CRC sensitivity and specificity were used for stool-based tests. Adherence was set at (1) 100%, (2) 30%–70%, in 10% increments, and (3) real-world rates for stool-based tests (mt-sDNA = 65.6%; FIT = 42.6%; FOBT = 34.4%). Assuming perfect adherence, a blood-based test produced ≥19 lower life-years gained (LYG) than stool-based strategies. At the best-case scenario for blood-based tests (100% adherence and 20% adenoma sensitivity), mt-sDNA at real-world adherence achieved more LYG (287.2 vs. 297.1, respectively) with 14% fewer colonoscopies. At 100% blood-based test adherence and real-world mt-sDNA and FIT adherence, the blood-based test would require advanced adenoma sensitivity of 30% to reach the LYG of mt-sDNA (297.1) and ∼15% sensitivity to reach the LYG of FIT (258.9). This model suggests that blood-based tests with CMS minimally acceptable CRC sensitivity and low advanced adenoma sensitivity will frequently yield inferior outcomes to stool-based testing across a wide range of adherence assumptions.

## Introduction

In 2021, there were an estimated 150,000 new cases of colorectal cancer (CRC) in the United States and an estimated 53,000 CRC-related deaths.^1^ Fortunately, CRC screening has been demonstrated to effectively reduce disease-related incidence and mortality through earlier detection.^2,3^ Broadly endorsed CRC screening options include relatively invasive tests, such as colonoscopy, that can identify and remove some colorectal neoplasia during the initial examination.^4–6^ Colonoscopies have high sensitivity and specificity to detect adenomas and CRC, but require bowel cleansing and often use sedation.

Alternatively, noninvasive stool-based screening tests are taken at home without the pretest preparation or risks associated with colonoscopy (eg, colon perforation). Stool-based tests, including fecal immunochemical test (FIT), fecal occult blood test (FOBT), and multitarget stool DNA (mt-sDNA), detect markers released from both CRCs and precancerous adenomas^7^ and are recommended for average-risk screening by the US Preventive Services Task Force (USPSTF), American Cancer Society, and other major guidelines.^4–6^ All positive noninvasive stool-based screening tests should be followed up with colonoscopy.^4–6^ Blood-based biomarker tests represent a newer category of noninvasive CRC screening, but they are currently not recommended in major guidelines as first-line options.^4–6^

The Centers for Medicare & Medicaid Services (CMS) recommend covering blood-based biomarker tests with proposed minimum performance (sensitivity and specificity) thresholds for CRC screening test every 3 years for average-risk, asymptomatic Medicare beneficiaries of ages 50–85 years.^8^ The proposed minimum performance thresholds are 74% for CRC sensitivity and 90% for CRC specificity compared with colonoscopy. These thresholds are based on the minimum sensitivity and specificity for FIT and mt-sDNA.^8^ Interestingly, the recently issued CMS guidance does not stipulate performance thresholds for the detection of precancerous adenomas, a critical outcome measure that substantially impacts the effectiveness of screening modalities.^8^

To provide further clarity regarding the potential clinical utility of blood-based CRC screening under a number of potential clinical scenarios, microsimulation modeling analyses were conducted to predict life-years gained (LYG), CRC incidence, and CRC mortality outcomes resulting from blood-based CRC screening based on CMS minimum thresholds. These outcomes were then compared with available stool-based screening options (FIT, FOBT, and mt-sDNA).

## Methods

### Microsimulation model

**FIG. 1.**
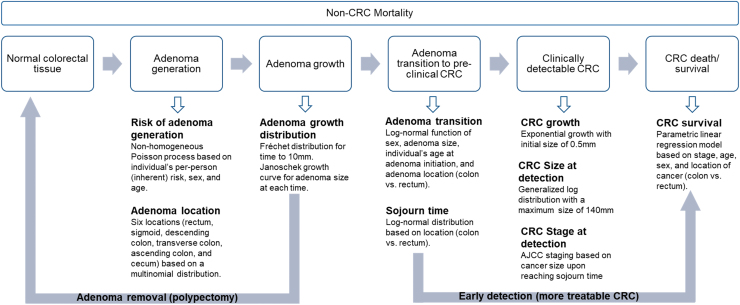
CRC-AIM microsimulation. AJCC, American Joint Committee on Cancer; CRC, colorectal cancer.

Colorectal Cancer and Adenoma Incidence and Mortality Microsimulation Model (CRC-AIM) is a microsimulation model that incorporates assumptions for the known and unknown natural history of CRC and for CRC screening test parameters to simulate the impact of screening on CRC outcomes (Fig. 1).^9,10^ CRC-AIM has been previously validated to US CRC incidence and mortality presented in the Surveillance, Epidemiology, and End Results (SEER) database and cross-model validated against CRC Cancer Intervention and Surveillance Modeling Network (CISNET) models (ie, CRC-SPIN, SimCRC, and MISCAN)^11–14^ that have been widely used for USPSTF^15,16^ and American Cancer Society^5^ guideline recommendations.

The natural history component of the model follows the course of CRC using an adenoma–carcinoma sequence. The assumptions related to the natural history used in CRC-AIM have been previously described and include adenoma generation, adenoma growth, transition chance to preclinical CRC, CRC growth to become clinically detectable, and CRC death or survival, as shown in Figure 1.^10^

The CRC screening test component of CRC-AIM incorporates sensitivity and specificity of each modality, the frequency each test is used, adherence to the assigned screening frequency, and age interval for screening.^9^ It is assumed that when a screening test is positive, either any identified precancerous adenomas are removed to prevent the progression to preclinical CRC or that detection of early stage CRC is potentially more treatable, whereby mortality is reduced (Fig. 1).

### Screening test performance assumptions

CRC-AIM was used to simulate outcomes of blood- and stool-based tests (FIT, FOBT, and mt-sDNA) for average-risk individuals free of diagnosed CRC at age 40 years and screened between ages 45 and 75 years. The age range of 45–75 years was selected per USPSTF findings that CRC screening among such individuals provides substantial or moderate net benefit.^4^ Per CMS proposed criteria, CRC sensitivity and specificity for a blood-based test were set at 74% and 90%, respectively.^8^ Since CMS did not propose thresholds for adenoma sensitivity for a blood-based test, adenoma sensitivity was varied in 5 scenarios ranging from 10% to 20% (Table 1).

**Table 1.** Screening Test Assumptions

Screening test, scenario	Sensitivity			Specificity	Screening interval
	Adenoma		CRC		
	<10 mm	≥10 mm			
mt-sDNA	15%^19^	42%^19,a^	94%^19^	91%^19^	3 Years
FIT	7%^19^	22%^19,a^	74%^19^	97%^19^	1 Year
FOBT	5%^18^	11%^18^	68%^18^	97%^18^	1 Year
Blood, S1	10%	10%	74%^8^	90%^8^	3 Years
Blood, S2	12.5%	15%	74%	90%	3 Years
Blood, S3	15%	15%	74%	90%	3 Years
Blood, S4	15%	20%	74%	90%	3 Years
Blood, S5	20%	20%	74%	90%	3 Years

^a^ Sensitivity for persons with advanced adenomas (ie, ≥10 mm or adenomas with advanced history); sensitivity was not reported for the subset of persons with ≥10 mm adenomas separately from advanced adenomas.

CRC, colorectal cancer; FIT, fecal immunochemical test; FOBT, fecal occult blood test; mt-sDNA, multitarget stool DNA.

The lower sensitivity of 10% is the false-positive rate of the CMS minimum threshold for specificity, and the maximum sensitivity of 20% is based on preliminary clinical results for a blood-based test.^17^ In 2 of the scenarios, adenoma sensitivity was assumed to be different for nonadvanced (<10 mm) and advanced (≥10 mm) adenomas (Table 1). There is currently not enough evidence for blood-based test adenoma sensitivity based on adenoma size, so it was assumed that the adenoma detection for a nonadvanced adenoma was half that of a large adenoma after excluding an accidental finding. Adenoma and CRC sensitivity and specificity for each stool-based test were those used by the USPSTF in their 2021 updated recommendations (Table 1).^18,19^

### Screening test strategy assumptions

FIT and FOBT were recommended to be completed every year. The blood-based test and mt-sDNA were recommended to be completed every 3 years. For the primary analysis, adherence to screening intervals was assumed to be 100%. For secondary analysis, adherence was examined between 30% and 70%, in 10% increments, or at reported real-world adherence rates of 65.6% for mt-sDNA,^20^ 42.6% for FIT,^21^ and 34.4% for FOBT.^21^ Adherence to follow-up colonoscopies after a positive stool- or blood-based test was assumed to be 100%.

### Outcomes

Estimated outcomes were LYG and reductions in CRC incidence and mortality compared with no screening. Outcomes were per 1000 individuals.

### Sensitivity analysis

To determine the threshold for adenoma sensitivity that would match outcomes with the stool-based tests, sensitivity analyses with >20% nonadvanced (<10 mm) and advanced (≥10 mm) adenoma sensitivities for the blood-based test were conducted.

### Data availability

Data were generated by the authors and are available on request. To promote full transparency and credibility of the CRC-AIM model, its formulas and parameters are available on a public repository (https://github.com/CRCAIM/CRC-AIM-Public).

## Results

**FIG. 2.**
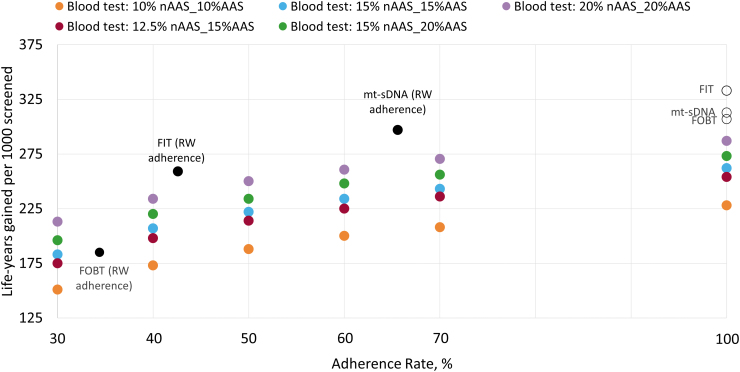
LYG by adherence rate per 1000 patients over lifetime horizon. AAS, advanced adenoma (≥10 mm) sensitivity; FIT, fecal immunochemical test; FOBT, fecal occult blood test; LYG, life-years gained; mt-sDNA, multi-target stool DNA; nAAS, nonadvanced adenoma (<10 mm) sensitivity; RW, real world.

In the scenario where a blood-based test meets CMS minimum performance thresholds on sensitivity and specificity and with perfect adherence assumed for all tests, a blood-based test produced ≥19 lower estimated LYG than currently available stool-based tests (Fig. 2; Supplementary Table S1). When the *best-case* scenario for blood-based tests was modeled (100% adherence *and* 20% nonadvanced and advanced adenoma sensitivity) and compared with real-world adherence rates for stool-based tests, mt-sDNA achieved more LYG (287.2 vs 297.1, respectively; Fig. 2) with 14% fewer total colonoscopies (1601.9 vs 1404.5, respectively; Supplementary Table S1).

High adherence (>60%) and high nonadvanced and advanced adenoma sensitivity (both 20%) are needed for the blood-based test to exceed the LYG of FIT at real-world adherence (Fig. 2) with 40% higher total colonoscopies (1409.7 vs 1004.9, respectively; Supplementary Table S1). Using real-world adherence for FOBT, the blood-based test produces more LYG in most of the adherence and sensitivity scenarios (Fig. 2).

Sensitivity analyses were conducted to determine what threshold of adenoma sensitivity would be required by a blood-based test to reach the same outcomes as mt-sDNA. At 100% adherence for the blood-based test and real-world adherence for mt-sDNA (65.6%), the blood-based test would require nonadvanced adenoma sensitivity of 20% and advanced adenoma sensitivity of 30% to reach the LYG of mt-sDNA (297.1; Supplementary Table S2). At real-world adherence for FIT (42.6%), ∼15% sensitivity for both nonadvanced and advanced adenomas would be required for the blood-based test to reach the LYG of FIT (258.9; Supplementary Table S1).

**FIG. 3.**
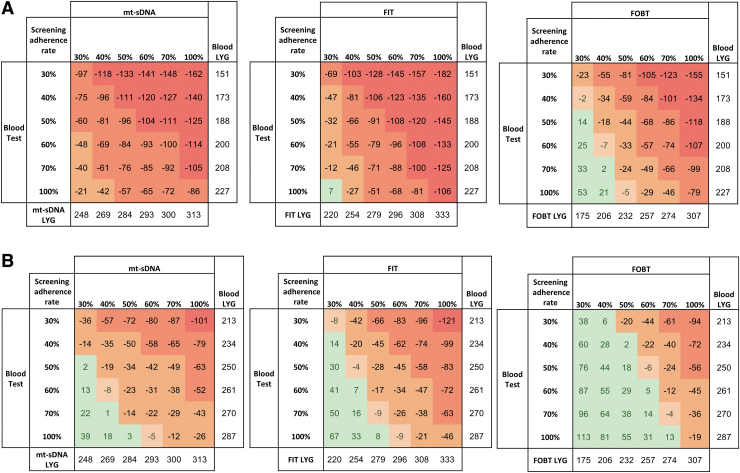
LYG per 1000 patients screened over lifetime horizon based on different adherence rates for blood-based test adenoma sensitivity at **(A)** 10% and **(B)** 20%. Numbers represent ranges of incremental LYG versus the blood-based test. A positive value (green) indicates the stool-based test has less LYG than the blood-based test, and a negative value (red/orange) indicates the stool-based test has more LYG than the blood-based test. Adherence rates ranged from 100% or 30%–70% for both stool- and blood-based tests.

Figure 3 illustrates the tradeoffs in LYG produced between a blood-based test and each of the 3 stool-based screening tests across a broad range of adherence rates. In a scenario wherein a blood-based test has 10% sensitivity for both nonadvanced (<10 mm) *and* advanced (≥10 mm) adenomas, mt-sDNA had at least 21 higher LYG (Fig. 3A) and at least 11% and 7% higher reductions in absolute CRC incidence and mortality per 1000 individuals, respectively, at all explored adherence rates (Supplementary Figs. S1A and S2A).

When the blood-based test adenoma sensitivity for both small and advanced adenomas was increased to 20%, mt-sDNA and FIT had higher LYG (Fig. 3B), and mt-sDNA had higher reductions in incidence and mortality, at identical adherence (Supplementary Figs. S1B and S2B).

## Discussion

The results of this microsimulation model indicate that estimated LYG are inferior with a blood-based test that meets CMS minimum performance standards and interval testing recommendations as compared with currently endorsed stool-based screening strategies when adherence to all tests is assumed to be 100%. Using real-world adherence rates for stool-based tests, mt-sDNA produced more LYG and fewer colonoscopies than the blood-based test with 100% adherence and best-case adenoma sensitivity. To exceed the LYG of FIT at real-world adherence, a blood-based test needed high adenoma detection and adherence. In contrast, the blood-based test produced more LYG than FOBT at real-world adherence.

The main driver of the scenarios wherein the blood-based test was inferior to the stool-based tests was the inability of the blood-based test to sufficiently detect advanced adenomas. Other models have also demonstrated the important contribution of the detection and removal of advanced adenomas in reducing CRC incidence and mortality.^15^ For this analysis, adenoma sensitivity for the blood-based test was assumed to be between 10% and 20%, producing a wide range of LYG (10%, 228; 20%, 287) given perfect adherence. The sensitivity assumptions of the stool-based tests to detect adenomas (42% mt-sDNA, 22% FIT, and 11% FOBT) were the same as those used by the USPSTF in their 2021 recommendations^18^ and were derived from data compiled from clinical studies.^19^ In their assessment of blood-based tests for CRC screening,^8^ CMS identified 2 studies that reported advanced adenoma sensitivity.^22,23^ These 2 studies used samples from the same patient population, but used first- and second-generation tests for which sensitivity was reported as 11% and 22%, respectively.^22,23^ In addition, preclinical results released for another blood-based test reported an advanced adenoma sensitivity of 20%,^17^ although results reported in December 2022 from the ECLIPSE trial indicate an advanced adenoma sensitivity of 13% for this blood test.^24^ Head-to-head trials will be the only way to determine whether the advanced adenoma sensitivity for a blood-based test is truly less than for stool-based tests. Therefore, for the current analysis, a lower limit for adenoma sensitivity was set at 10%, which is the false-positive rate of the CMS minimum threshold for specificity (essentially 0% adenoma sensitivity), and an additional range of adenoma sensitivities were applied up to a maximum of 20% to account for the full range of sensitivities at which the available blood-based tests are currently capable. The sensitivity analysis indicates that even when comparing the blood-based test at 100% adherence with mt-sDNA at real-world adherence, the blood-based test would require advanced adenoma sensitivity of 30% to achieve comparable clinical outcomes.

Screening for CRC needs to be repeated at intervals that vary depending on the screening test. Perfect adherence to the recommended 1- or 3-year intervals for noninvasive screening over the course of 30 years is not realistic in routine clinical practice. Thus, published real-world adherence rates of 65.6% for mt-sDNA,^20^ 42.6% for FIT,^21^ and 34.4% for FOBT^21^ were applied in the model. Other adherence rates for the stool-based tests have been published that vary by the population evaluated and how adherence was defined.^25–32^ Therefore, a range of adherence rates from 30% to 100% were also applied in the model to accommodate the potential variability in real-world adherence.

Higher LYG with mt-sDNA were observed compared with the blood-based test (with 10% adenoma sensitivity) at all levels of adherence. When the levels of adherence were identical between the tests, mt-sDNA and FIT maintained an advantage in LYG over the blood-based test at the highest assumed adenoma sensitivity (20% for nonadvanced and advanced adenomas). The blood-based test at 20% adenoma sensitivity outperformed FOBT at lower adherence rates. The adherence to a triennial blood-based CRC screening test is unknown, but US self-reported screening surveillance data indicated that in 2009, 89% of adults aged 45–64 years in the general population had undergone blood cholesterol screening in the previous 5 years.^33^ The results of a randomized controlled trial found that offering a blood test as a second-line screening option to patients who had previously declined FIT or a colonoscopy increased initial screening from 9.6% in the control group (reoffered FIT or colonoscopy) to 17.1% in the intervention group (offered blood test as second-line option).^34^ The rate of completing a full screening strategy (eg, follow-up colonoscopy) was not significantly different between the 2 groups. In contrast, another randomized controlled trial found that when nonparticipants of a previous CRC screening trial were offered FIT, a blood test, or a choice between the 2, there was no difference in participation rates among the groups and FIT was preferred in the choice group.^35^ With respect to the influence of screening adherence, 1 possible scenario is that real-world adherence to a blood-based test strategy will be higher than to a stool-based test strategy. Adherence may also depend on whether the blood-based test is recommended annually or triennially. In the current analysis, higher LYG with triennial mt-sDNA were observed compared with the triennial blood-based test (with 10% adenoma sensitivity), even when assuming adherence as low as 30% for mt-sDNA and as high as 100% for the blood-based test.

A limitation of this analysis is that the sensitivity of the blood- and stool-based screening tests to detect sessile serrated polyps (SSPs) was not specifically modeled. SSPs develop from a different genetic pathway than the conventional adenoma pathway, and ∼20%–30% of CRCs arise from this alternative pathway.^36^ The sensitivity to detect SSPs varies among CRC screening tests. In 1 study, mt-sDNA was more sensitive than FIT to detect SSPs measuring ≥10 mm (42.4% vs 5.1%, respectively; *P* < 0.001).^37^ The sensitivity of blood-based tests to detect SSPs is unknown. Another limitation is that the current analysis was limited to evaluation of a 3-year interval for blood-based tests, in alignment with the recently proposed CMS guidance. Assessment of additional intervals may be informative and could be considered for future analyses.

In sum, data from this modeling study suggest that blood-based screening strategies leveraging tests with minimally acceptable sensitivity for CRC, coupled with low sensitivity for adenomas (using previously reported estimates), will yield clinical outcomes that are frequently inferior to stool-based strategies, even across a wide range of adherence assumptions. Clinical trials may yield different results than the outcomes simulated in this analysis, and head-to-head randomized trials are needed for verification. To realize meaningful public health benefits of CRC screening, strengths and weaknesses of various screening modalities must be conveyed to patients.

## Supplementary Material

Supplemental data

Supplemental data

Supplemental data

Supplemental data
