# Supplementary material for: Comparison of Simulated Outcomes Between Stool- and Blood-Based Colorectal Cancer Screening Tests
Source: Popul Health Manag. 2023 Aug 14;26(4):239–45. doi: 10.1089/pop.2023.0037 (PMC10457617; doi:10.1089/pop.2023.0037)
Supplement: Supplemental data [file Suppl_TableS1.docx]

**Table S1**. Estimated outcomes with blood-based and stool-based test under various adherence and blood-based test adenoma sensitivity scenarios.

| **Screening Test** | **Adherence** | **Total COLs** | **CRC cases** | **CRC deaths** | **LY with CRC** | **LYG** | **CRC incidence reduction** | **CRC mortality reduction** |
| --- | --- | --- | --- | --- | --- | --- | --- | --- |
| **No screening** | -- | 80.1 | 80.1 | 36.8 | 643.1 | 0.000 | 0.0% | 0.0% |
| **mt-sDNA** | **30%** | 1084.2 | 40.1 | 15.5 | 402.8 | 248.1 | 50.0% | 57.8% |
|  | **40%** | 1208.4 | 36.6 | 13.9 | 375.1 | 269.1 | 54.3% | 62.2% |
|  | **50%** | 1299.0 | 34.3 | 12.8 | 356.2 | 284.1 | 57.2% | 65.3% |
|  | **60%** | 1371.7 | 32.6 | 12.0 | 343.1 | 292.6 | 59.3% | 67.4% |
|  | **70%** | 1428.2 | 31.4 | 11.4 | 332.4 | 299.6 | 60.8% | 68.9% |
|  | **100%** | 1546.9 | 28.9 | 10.3 | 311.3 | 313.2 | 64.0% | 71.9% |
|  | **RW** | 1404.5 | 31.9 | 11.7 | 334.5 | 297.1 | 60.2% | 68.1% |
| **FIT** | **30%** | 811.4 | 46.7 | 18.2 | 466.4 | 220.2 | 41.8% | 50.5% |
|  | **40%** | 972.7 | 40.8 | 15.3 | 424.9 | 254.2 | 49.1% | 58.4% |
|  | **50%** | 1102.8 | 36.7 | 13.3 | 391.3 | 278.8 | 54.3% | 63.8% |
|  | **60%** | 1229.3 | 33.2 | 11.8 | 361.9 | 295.8 | 58.5% | 68.0% |
|  | **70%** | 1321.5 | 30.9 | 10.9 | 339.6 | 308.1 | 61.5% | 70.5% |
|  | **100%** | 1585.5 | 25.6 | 8.7 | 286.9 | 333.1 | 68.0% | 76.2% |
|  | **RW** | 1004.9 | 39.8 | 14.9 | 419.2 | 258.9 | 50.3% | 59.6% |
| **FOBT** | **30%** | 647.0 | 57.2 | 22.6 | 562.8 | 174.5 | 28.6% | 38.7% |
|  | **40%** | 794.6 | 52.1 | 19.7 | 534.4 | 206.4 | 35.0% | 46.5% |
|  | **50%** | 923.9 | 47.7 | 17.5 | 507.1 | 232.3 | 40.5% | 52.3% |
|  | **60%** | 1040.4 | 44.1 | 15.6 | 485.1 | 256.6 | 44.9% | 57.6% |
|  | **70%** | 1148.8 | 40.8 | 14.2 | 457.0 | 273.9 | 49.0% | 61.3% |
|  | **100%** | 1426.6 | 33.8 | 11.2 | 392.8 | 306.6 | 57.8% | 69.5% |
|  | **RW** | 698.3 | 55.3 | 21.6 | 551.5 | 185.1 | 30.9% | 41.4% |
| **Blood, S1**  **nAAS=10% AAS=10%** | **30%** | 820.6 | 60.0 | 24.3 | 576.1 | 151.3 | 25.1% | 34.0% |
|  | **40%** | 938.5 | 57.0 | 22.5 | 563.0 | 172.9 | 28.9% | 38.9% |
|  | **50%** | 1030.0 | 54.6 | 21.2 | 548.5 | 188.1 | 31.9% | 42.3% |
|  | **60%** | 1099.6 | 53.0 | 20.3 | 541.7 | 199.6 | 33.9% | 44.9% |
|  | **70%** | 1157.2 | 51.7 | 19.6 | 535.7 | 208.0 | 35.5% | 46.7% |
|  | **100%** | 1285.8 | 48.7 | 17.9 | 519.1 | 227.5 | 39.2% | 51.3% |
| **Blood, S2 nAAS=12.5% AAS= 15%** | **30%** | 920.4 | 54.0 | 21.9 | 523.3 | 175.0 | 32.6% | 40.6% |
|  | **40%** | 1043.3 | 50.8 | 20.0 | 506.8 | 197.9 | 36.6% | 45.6% |
|  | **50%** | 1146.0 | 48.1 | 18.6 | 487.9 | 214.4 | 40.0% | 49.4% |
|  | **60%** | 1216.9 | 46.5 | 17.8 | 479.3 | 224.5 | 42.0% | 51.7% |
|  | **70%** | 1275.4 | 44.8 | 16.8 | 468.1 | 235.8 | 44.1% | 54.2% |
|  | **100%** | 1405.9 | 41.7 | 15.3 | 447.3 | 253.8 | 48.0% | 58.4% |
| **Blood, S3 nAAS=15% AAS= 15%** | **30%** | 967.2 | 52.3 | 21.1 | 509.2 | 183.4 | 34.7% | 42.7% |
|  | **40%** | 1101.9 | 48.5 | 19.1 | 487.0 | 206.8 | 39.5% | 48.0% |
|  | **50%** | 1194.8 | 46.1 | 17.9 | 471.0 | 221.6 | 42.5% | 51.4% |
|  | **60%** | 1268.3 | 43.9 | 16.8 | 456.0 | 234.1 | 45.2% | 54.5% |
|  | **70%** | 1339.8 | 42.3 | 16.0 | 445.9 | 242.9 | 47.2% | 56.4% |
|  | **100%** | 1470.2 | 39.3 | 14.4 | 426.3 | 262.4 | 50.9% | 60.8% |
| **Blood, S4**  **nAAS=15% AAS= 20%** | **30%** | 1003.7 | 49.1 | 19.8 | 479.1 | 196.4 | 38.7% | 46.1% |
|  | **40%** | 1134.9 | 45.6 | 17.9 | 460.1 | 219.7 | 43.1% | 51.4% |
|  | **50%** | 1239.0 | 43.0 | 16.7 | 439.5 | 234.4 | 46.3% | 54.6% |
|  | **60%** | 1316.4 | 40.9 | 15.5 | 424.0 | 247.7 | 49.0% | 57.8% |
|  | **70%** | 1369.6 | 39.5 | 14.9 | 414.3 | 256.3 | 50.7% | 59.4% |
|  | **100%** | 1505.7 | 36.4 | 13.4 | 391.7 | 272.9 | 54.5% | 63.7% |
| **Blood, S5 nAAS=20% AAS= 20%** | **30%** | 1089.2 | 45.5 | 18.2 | 451.3 | 212.5 | 43.2% | 50.5% |
|  | **40%** | 1231.3 | 41.8 | 16.4 | 425.7 | 234.1 | 47.8% | 55.5% |
|  | **50%** | 1334.8 | 39.1 | 15.0 | 407.3 | 250.4 | 51.2% | 59.2% |
|  | **60%** | 1409.7 | 37.3 | 14.2 | 392.4 | 261.4 | 53.5% | 61.5% |
|  | **70%** | 1472.6 | 35.7 | 13.4 | 380.2 | 270.2 | 55.4% | 63.5% |
|  | **100%** | 1601.9 | 32.7 | 11.9 | 358.2 | 287.2 | 59.2% | 67.6% |

AAS, advanced adenoma (≥ 10 mm) sensitivity; COL, colonoscopy; CRC, colorectal cancer; FIT, fecal immunochemical test; FOBT, fecal occult blood test; LY, life-years; LYG, life-years gained; mt-sDNA, multi-target stool DNA; nAAS, non-advanced adenoma (<10 mm) sensitivity; RW, real-world; S, scenario.
