# Supplementary material for: Comparison of Simulated Outcomes Between Stool- and Blood-Based Colorectal Cancer Screening Tests
Source: Popul Health Manag. 2023 Aug 14;26(4):239–45. doi: 10.1089/pop.2023.0037 (PMC10457617; doi:10.1089/pop.2023.0037)
Supplement: Supplemental data [file Suppl_TableS2.docx]

**Table S2.** Estimated outcomes of sensitivity analyses with blood-based test under various adherence and expanded blood-based test adenoma sensitivity scenarios.

| **Blood-test sensitivity** | **Adherence** | **Total COLs** | **CRC cases** | **CRC deaths** | **LYG** | **CRC Incidence reduction** | **CRC Mortality reduction** |
| --- | --- | --- | --- | --- | --- | --- | --- |
| **nAAS=20%**  **AAS= 30%** | 30% | 1147.4 | 41.4 | 16.5 | 231.3 | 48.3% | 55.1% |
|  | 40% | 1279.0 | 37.7 | 14.7 | 252.6 | 53.0% | 59.9% |
|  | 50% | 1382.9 | 35.0 | 13.5 | 268.5 | 56.4% | 63.3% |
|  | 60% | 1462.4 | 33.0 | 12.6 | 278.8 | 58.8% | 65.8% |
|  | 70% | 1521.6 | 31.6 | 11.9 | 287.4 | 60.6% | 67.7% |
|  | 100% | 1647.9 | 29.0 | 10.9 | 300.7 | 63.8% | 70.5% |
| **nAAS=30%**  **AA= 30%** | 30% | 1288.7 | 36.1 | 14.4 | 253.1 | 54.9% | 60.8% |
|  | 40% | 1426.9 | 32.3 | 12.6 | 272.6 | 59.7% | 65.7% |
|  | 50% | 1526.3 | 29.9 | 11.5 | 287.9 | 62.7% | 68.8% |
|  | 60% | 1602.3 | 28.0 | 10.7 | 298.4 | 65.0% | 71.0% |
|  | 70% | 1665.2 | 26.9 | 10.1 | 305.6 | 66.4% | 72.6% |
|  | 100% | 1788.0 | 24.6 | 9.1 | 317.9 | 69.3% | 75.2% |
| **nAAS=25%**  **AAS= 40%** | 30% | 1263.3 | 35.3 | 14.2 | 256.7 | 55.9% | 61.5% |
|  | 40% | 1399.2 | 31.7 | 12.4 | 278.6 | 60.4% | 66.3% |
|  | 50% | 1499.4 | 29.2 | 11.2 | 293.3 | 63.5% | 69.5% |
|  | 60% | 1568.1 | 27.8 | 10.7 | 300.3 | 65.3% | 71.0% |
|  | 70% | 1629.4 | 26.7 | 10.1 | 307.5 | 66.7% | 72.6% |
|  | 100% | 1747.3 | 24.5 | 9.2 | 319.8 | 69.4% | 75.1% |
| **nAAS=40%**  **AAS= 40%** | 30% | 1430.8 | 29.5 | 11.8 | 281.9 | 63.2% | 68.1% |
|  | 40% | 1563.1 | 26.2 | 10.1 | 301.7 | 67.3% | 72.4% |
|  | 50% | 1662.6 | 23.9 | 9.2 | 313.7 | 70.1% | 74.9% |
|  | 60% | 1731.1 | 22.6 | 8.6 | 320.3 | 71.8% | 76.5% |
|  | 70% | 1790.6 | 21.8 | 8.2 | 325.9 | 72.8% | 77.6% |
|  | 100% | 1905.7 | 20.3 | 7.5 | 336.7 | 74.7% | 79.5% |

AAS, advanced adenoma (≥ 10 mm) sensitivity; COL, colonoscopy; CRC, colorectal cancer; LYG, life-years gained; nAAS, non-advanced adenoma (<10 mm) sensitivity.
