# Supplementary material for: Comparison of Simulated Outcomes Between Stool- and Blood-Based Colorectal Cancer Screening Tests
Source: Popul Health Manag. 2023 Aug 14;26(4):239–45. doi: 10.1089/pop.2023.0037 (PMC10457617; doi:10.1089/pop.2023.0037)
Supplement: Supplemental data [file Suppl_FigS2.docx]

**Figure S2.** Colorectal cancer mortality reduction (MR) per 1,000 patients screened over lifetime horizon based on different adherence rates for blood-based test adenoma sensitivity at A) 10% and B) 20%. Numbers represent ranges of incremental MR versus the blood-based test. A positive value (green) indicates the stool-based test has less MR than the blood-based test, and a negative value (red/orange) indicates the stool-based test has more MR than the blood-based test. Adherence rates ranged from 100% or 30-70% for both stool- and blood-tests. FIT, fecal immunochemical test; FOBT, fecal occult blood test; mt-sDNA, multi-target stool DNA.

**A)**

**B)**
